# A Large Case-Control Study Performed in Spanish Population Suggests That *RECQL5* Is the Only RECQ Helicase Involved in Breast Cancer Susceptibility

**DOI:** 10.3390/cancers14194738

**Published:** 2022-09-28

**Authors:** Erik Michel Marchena-Perea, Milton Eduardo Salazar-Hidalgo, Alicia Gómez-Sanz, Mónica Arranz-Ledo, Alicia Barroso, Victoria Fernández, Hugo Tejera-Pérez, Guillermo Pita, Rocío Núñez-Torres, Luz Pombo, Rafael Morales-Chamorro, Juana María Cano-Cano, Maria del Carmen Soriano, Pilar Garre, Mercedes Durán, María Currás-Freixes, Miguel de la Hoya, Ana Osorio

**Affiliations:** 1 Human Cancer Genetics Programme, Familial Cancer Clinical Unit, Spanish National Cancer Research Centre (CNIO), 28029 Madrid, Spain; 2 Molecular Oncology Laboratory (CIBERONC), Hospital Clínico San Carlos, IdISSC, 28040 Madrid, Spain; 3 Cancer Genetics Group, Unidad de Excelencia Instituto de Biología y Genética Molecular, Universidad de Valladolid-Consejo Superior de Investigaciones Científicas (IBGM, UVa-CSIC), 47003 Valladolid, Spain; 4 Human Cancer Genetics Programme, Human Genotyping Unit (CEGEN), Spanish National Cancer Research Centre (CNIO), 28029 Madrid, Spain; 5 Medical Oncology Section, Universitary Hospital Complex of Albacete, 02006 Albacete, Spain; 6 Medical Oncology Section, Hospitalary Compex La Mancha Centro, 13600 Alcázar de San Juan, Spain; 7 Medical Oncology Service, Universitary General Hospital of Ciudad Real, 13005 Ciudad Real, Spain; 8 Oncology Service, Virgen de la Luz Hospital, 16002 Cuenca, Spain; 9 Spanish Network on Rare Diseases (CIBERER), 28029 Madrid, Spain; 10 Genetics Service, Fundación Jiménez Díaz, 28043 Madrid, Spain

**Keywords:** breast cancer (BC), RECQ helicase family, next-generation sequencing, BRCAX

## Abstract

**Simple Summary:**

Around 50% of the familial breast cancer (BC) cases are estimated to be caused by variants in low-, moderate-, and high-risk susceptibility genes; however, the other half is of unknown origin. The finding of new susceptibility genes is key to improve diagnosis, take preventive measures, and identify new therapies. In this context, previous studies have discussed whether the genes encoding for the RECQ helicase family could play a role in BC susceptibility, without very conclusive results. To clarify this, in this study, we sequenced the whole coding sequence of the *RECQL1*, *BLM*, *WRN*, *RECQL4*, and *RECQL5* genes in 1993 Spanish BC familial cases and compared it with controls from gnomAD. No association was found for *RECQL1*, *BLM*, *WRN*, and *RECQL4*; however, we did find an association between *RECQL5* and breast cancer as a moderate-risk gene, making it a perfect candidate for further studies.

**Abstract:**

Around 50% of the familial breast cancer (BC) cases are estimated to be caused by germline variants in known low-, moderate-, and high-risk susceptibility genes, while the other half is of unknown genetic origin. In the present study, we wanted to evaluate the role of the RECQ helicases, some of which have been studied in the past as candidates, with unclear results about their role in the disease. Using next-generation sequencing (NGS) technology, we analyzed the whole coding sequence of *BLM*, *RECQL1*, *RECQL4*, *RECQL5*, and *WRN* in almost 2000 index cases from BC Spanish families that had previously tested negative for the known BC susceptibility genes (BRCAX) and compared the results with the controls extracted from gnomAD. Our results suggest that *BLM*, *RECQL1*, *RECQL4*, and *WRN* do not play a major role in BC susceptibility. However, in the combined analysis, joining the present results with those previously reported in a series of 1334 BC Spanish patients and controls, we found a statistically significant association between Loss of Function (LoF) variants in *RECQL5* and BC risk, with an OR of 2.56 (*p* = 0.009; 95% CI, 1.18–4.98). Our findings support our previous work and places the *RECQL5* gene as a new moderate-risk BC gene.

## 1. Introduction

Breast cancer (BC) is the most common malignancy in women worldwide, as well as the second cause of death in women [1], with around 10–15% of the cases showing a familial aggregation of the disease [2]. Since their discovery, *BRCA1* and *BRCA2* are still the main high-risk susceptibility genes implicated in hereditary breast and ovarian cancer (HBOC). However, a few more moderate-to-high-risk genes, such as *ATM*, *BARD1*, *CHEK2*, *PALB2*, *RAD51C*, *RAD51D*, and *TP53* have been also established as bona-fide BC susceptibility genes [3], most of them taking part in DNA repair/maintenance pathways. Nevertheless, even with the advances of next-generation sequencing (NGS), in around 50% of the familial BC cases, the cause of the susceptibility is still undefined [4], highlighting the remarkable and complex genetic heterogeneity underlying this disease [5].

Since the appearance of exome sequencing, different genes have been pointed out as possible new susceptibility factors, but without very conclusive results [6]. In this context, the members of the RECQ helicase family (*BLM*, *RECQL1*, *RECQL4*, *RECQL5*, and *WRN*), some of them related to aging and/or cancer predisposition syndromes [7,8,9,10], have risen up in the last years as putative BC susceptibility genes. The RECQ helicase family participates in different DNA-related pathways, including in replication, base-excision repair, homologous recombination, transcription, telomere maintenance, and mitochondrial function [10]. The relevance of these genes in cell maintenance, and especially in DNA repair, makes them good candidates to be studied as putative BC susceptibility genes; however, there is lack of information or uncertainty about their role in the disease.

Remarkably, *RECQL1*, was proposed in 2015 as a new BC susceptibility gene by two independent studies [11,12]. However, subsequent studies have not confirmed its relationship with the disease, including the largest BC case-control study published so far, analyzing more than 113,000 women, in which no association between pathogenic variants in *RECQL1* and BC was found [3,13,14]. Taking this into account, there is conflict about whether it should be considered as a susceptibility gene [15]. Interestingly, biallelic mutations in *RECQL1* have been just associated with the new genome instability disorder, RECON syndrome [16].

*BLM* was also proposed in 2012 as a putative BC susceptibility gene in the Russian population, where the pathogenic variant c.1642C>T; p.Gln548Ter was repeatedly found in BC cases [17], a finding that was supported by another study of in Slavic populations one year later [18]. The role of *BLM* in various steps of the homologous recombination pathway [19] had positioned it as a perfect candidate BC susceptibility gene; however, as with RECQL1, there are no conclusive results about its role in the disease, since subsequent studies have not supported the initial findings [13,14,20,21,22,23]. In this regard, it is worth noting a recent study in which more than 14,000 patients and almost 5000 controls were analyzed, with no evidences of association of the founder mutation in *BLM* with BC susceptibility [23]. On the other hand, biallelic mutation in *BLM* is associated with Bloom syndrome, another chromosome-instability syndrome that includes, among other features, a higher predisposition for developing cancer [24].

Regarding *WRN*, germline biallelic mutations in the gene cause the autosomal-recessive disorder Werner Syndrome, which is associated with premature aging and cancer [25]. Previous publications tip the balance to suggest that *WRN* is a BC susceptibility gene [26,27], but they are all based on small cohorts, and there is a need to validate these results.

In the case of *RECQL4*, biallelic mutations in this gene are associated with three different, although overlapping, genetic disorders: Rothmund–Thomson syndrome, RAPADILINO syndrome, and Baller–Gerold syndrome, which are characterized by premature aging, growth retardation, and predisposition to cancers, among others [28]. So far, there is very little information about the possible role of the gene in BC susceptibility [29,30].

Finally, *RECQL5* is the only RECQ helicase that has not been associated with a recessive syndrome and, while being an excellent candidate from the functional point of view, highlighting its role in Homologous Recombination (HR) [10], there is only one study from our own group evaluating the role of germline mutations in the gene in BC susceptibility. In 2019, we carried out a genetic and functional analysis in 1334 BC Spanish patients and controls and found LoF or likely LoF variants in *RECQL5* in almost 1% of the cases (OR, 6.7; *p* = 0.07; 95% CI, 0.95–302.75), leading to the proposal that it could be a new BC-susceptibility candidate gene [31]. However, no subsequent studies have been performed to validate these findings.

This uncertainty or lack of information justifies the importance of further studies of the RECQ helicase family in larger cohorts. Here, we studied an independent series of 1993 BC Spanish patients and compared them with controls from the gnomAD database, aiming to clarify the role of the RECQ helicase family in the disease and, more specifically, to validate our previous findings regarding the *RECQL5* gene.

## 2. Materials and Methods

### 2.1. BRCAX Cases

A total of 1993 index cases from BRCAX families were included that had previously tested negative for pathogenic or likely pathogenic variants in the *BRCA1*, *BRCA2*, PALB2, ATM, CHEK2, BRIP1, RAD51C, and RAD51D genes, either with the NGS gene panel used here or with that described in Benito-Sánchez et al., 2022 [32]. Samples came from seven Spanish centers: Spanish National Cancer Center (CNIO) (Madrid, Spain), Hospital Clínico San Carlos (Madrid, Spain), Hospital General La Mancha Centro (Ciudad Real, Spain), Complejo Hospitalario Universitario de Albacete, Hospital General Universitario de Ciudad Real, Hospital Virgen de la Luz (Cuenca, Spain), and Laboratorio de Cáncer Hereditario of Instituto de Biología y Genética Molecular (Universidad de Valladolid, Valladolid, Spain). BRCAX families were selected based on a minimum criterion of the presence of at least one female BC patient diagnosed before the age of 40 or at least two female BC patients, with at least one of them diagnosed before the age of 50; however, families with more BC cases or younger ages of diagnosis were prioritized. All participants signed an informed consent approved by the Ethics Committee of Carlos III Institute of Health (Madrid, Spain).

### 2.2. Controls

For the purpose of this study, data from non-cancer European non-Finnish individuals was extracted from the Genome Aggregation Database (gnomAD v2.1.1) and were used as controls (https://gnomAD.broadinstitute.org/) (accessed on 3 March 2022) [33]. Only variants flagged as Loss of Function were taken into account for the case-control analysis. We ruled out the following LoF variants, flagged as Low-Confidence by the predictor Loss-of-Function Transcript Effect Estimator (LOFTEE), integrated in gnomAD: Low-Confidence Protein Loss of Function (LC_pLOF), which is applied to variants that are most likely not correctly classified; variants with NAGNAG flags, which are assigned to variants located in a NAGNAG site, where an alternative splicing could be generated, but it is predicted to be rescued by an in-frame acceptor site, leading to no LoF effect; and Low-Complexity Region (LCR) flags that are associated with variants in regions that contain repeats of single amino acids and are likely misreported due to polymerase errors. In addition, the frequency of individual variants in the Spanish general population was obtained from the Collaborative Spanish Variant Server (CSVS) (http://csvs.clinbioinfosspa.es/) (accessed on 8 March 2022) [34]. These local controls were not available to perform a case-control study, which did not allow us to check for population specific effects.

### 2.3. DNA Isolation

Maxwell RSC automated instrument (promega) was used to carry out genomic DNA extraction from peripheral blood samples, following the protocol of the manufacturer. Quantification of purified DNA was performed with Quant-iT PicoGreen dsDNA reagent (Invitrogen).

### 2.4. Next-Generation Sequencing

The RECQ helicase genes were analyzed within a larger capture-based in-house NGS panel of 25 genes, using probes designed and manufactured by IDT (Integrated DNA Technologies, Coralville, IA, USA) (a complete description of the panel is not provided, as it contains other non-published candidate genes). The Nextera Flex for Enrichment protocol from Illumina was used to prepare the DNA libraries, starting with a minimum of 50 ng in the pre-enrichment stage, following the manufacturer’s instructions. DNA/RNA UD indexes from IDT for Illumina were used for each 96-well plate. Quality and quantity confirmation of the resulting libraries was carried out on a 2100 Bioanalyzer system (Agilent, Santa Clara, CA, USA). Pools of 12 samples were prepared, adding 500 ng of each pre-enriched library for hybridization and capture steps, being able to process up to 192 samples in each sequencing run. The probe hybridization and enrichment was carried out by following the IDT protocol (xGenTM Hybridization and Wash Kit, xGen Predesigned Gene Capture Pools; xGen Universal Blockers-NXT Mix (IDT, Coralville, IA, USA) and KAPA HiFi HotStart ReadyMix (Roche, Basel, Switzerland)). Finally, 16 pools containing 192 samples were sequenced afterward via MiSeq technology, generating 121 base-pair long reads.

### 2.5. Bioinformatics Analysis and Variant Filtration

For the analysis, we used the Illumina Local Run Manager software with the DNA Enrichment module. FASTQ files generated by MiSeq Reporter software (Illumina, San Diego, CA, USA) were aligned with the reference sequence, and these alignments were used to create VCF files that were uploaded in VariantStudio 2.0 software (Illumina, San Diego, CA, USA) to carry out the variant filtering and prioritization process. All samples considered for the analysis had a read depth of at least 20× in 95% of the target regions, and approximately 50% of them had 50× in 95% of the target regions. The primary filters used for the prioritization of the variants were heterozygous state, missense, frameshift, stop gain/lost variants, and variants, which were predicted to affect splicing. Candidate variants were considered that fulfilled the following criteria: the variant appeared in a region with at least 10 reads, the alternative variant was in >20% of the reads, the population frequency was <0.1% in every gnomAD population and subpopulation, and there were no more than 50 heterozygous variants in total. Variants were directly classified as (likely) pathogenic/LoF if they produced a premature stop codon (PTC) or affected consensus splice-sites (+/−1, 2). It is important to note that, in the case of *RECQL5*, strictly, none of the variants could be classified as pathogenic or likely pathogenic following the ACGM guidelines, given that, despite generating a truncated protein, they would never fulfill the PVS1 criteria for pathogenicity, as the gene has not been already proven to be implicated in any disease (PVS1: null variants (nonsense, frameshift, canonical +/−1 or 2 splice sites, initiation codon, and single or multiexon deletion) in a gene where LoF is a known mechanism of disease [35]). Therefore, we named all variants generating a PTC detected in *RECQL5* as (likely) LoF. All protein truncating variants detected in the RECQ helicases were confirmed by Sanger sequencing (Table 1 and Table 2) (primers available upon request).

In addition, putative missense pathogenic/LoF variants were prioritized by using a combination of eight in silico predictors, namely SIFT (https://sift.bii.a-star.edu.sg/) (accessed on 24 Frebruary 2022), MUTASTER (http://www.mutationtaster.org/) (accessed on 24 Frebruary 2022), Polyphen-2 (http://genetics.bwh.harvard.edu/pph2/) (accessed on 24 February 2022), FATHMM (http://fathmm.biocompute.org.uk/) (accessed on 24 February 2022), SNP&Go (http://snps-and-go.biocomp.unibo.it/snps-and-go/) (accessed on 24 February 2022), Mutation Assessor (http://mutationassessor.org/r3/) (accessed on 24 February 2022), MUTPRED (http://mutpred.mutdb.org/) (accessed on 24 February 2022), and Condel (http://bg.upf.edu/fannsdb/) (accessed on 24 February 2022). The predict protein score (PPS) calculus by position (https://www.predictprotein.org/) (accessed on 24 February 2022), using the SNAP2 algorithm, was also taken into account. We considered variants as putative pathogenic/LoF when at least five of the predictors indicated pathogenicity. These prioritization criteria are based in others previously reported showing high sensitivity and specificity for the classification of missense variants in RAD51C [36] and *RECQL5* [31]. Furthermore, we analyzed missense and synonymous variants, using Franklin (Genoox) (https://franklin.genoox.com/clinical-db/home) (accessed on 25 February 2022) and Varsome (https://varsome.com) (accessed on 25 February 2022) programs, which classify variants by using the American College of Medical Genetics (ACMG) criteria [35]. If a variant analysis did not reach 5 out of 9 predictors, indicating pathogenicity, but Varsome and Franklin did, we considered it to be putatively pathogenic/LoF, as well.

**Table 1:** Pathogenic or likely pathogenic variants found in the *RECQL1*, *BLM*, *WRN*, and *RECQL4* genes in the 1993 cases sequenced.

Gene	Reference	Nucleotide Change ^a^	Protein Change	gnomAD ^c^	CSVS ^d^	Previously Found
***RECQL1***	NM_002907.3	c.84delT	p.Thr29ArgfsTer14	NR	NR	
***BLM***	NM_000057.2	c.53_56delCCAG	p.Ala18GlufsTer7	NR	NR	
		c.1933C>T	p.Gln645Ter	0.00008810	NR	[37]
***WRN***	NM_000553.6	c.205dupA	p.Ile69AsnfsTer2	NR	NR	
		c.979G>T	p.Gly327Ter	NR	NR	
		c.2604G>A	p.Trp868Ter	NR	NR	
		c.4013del	p.Leu1338*	NR	NR	
		c.4117_4120dupAGAT	p.Cys1374Ter	NR	NR	
***RECQL4***	NM_004260.4	c.320delA	p.Gln107ArgfsTer7	NR	NR	
		c.447dupC	p.Ser150Leufs*8	NR	NR	
		c.1048_1049delAG ^b^	p.Arg350GlyfsTer21	NR	NR	
		c.2161C>T	p.Arg721Ter	0.00002179	1/2093	[38]
		c.2269C>T	p.Gln757Ter	0.0001494	1/2093	[38]
		c.2547_2548del ^b^	p.(Phe850Profs*33)	NR	1/2093	[39]
		c.3217del	p.(Thr1073Profs*8)	NR	NR	

All variants were classified as pathogenic or likely pathogenic, following ACMG guidelines, according to the calculations made by the Franklin Genoox platform for variant interpretation (https://franklin.genoox.com/clinical-db/home). ^a^ Numbering starting at the “A” of the first ATG, following HGVS guidelines (www.hgvs.org/mutnomen). ^b^ Variants found twice in our series. ^c^ Allele frequency reported in gnomAD in non-cancer European non-Finnish individuals. NR: not reported. ^d^ Number of heterozygotes for the variant/total number of individuals reported in the Collaborative Spanish Variant Server (CSVS). Thompson E et al., 2012 [37]; Cao F. et al., 2017 [38]; Siitonen A et al., 2009 [39].

**Table 2:** LoF or likely LoF variants found in *RECQL5* in the 1993 cases sequenced.

Gene	Reference	Nucleotide Change	Protein Change	Phenotype ^b^	gnomAD	CSVS
***RECQL5***	NM_004259.6	c.130G>A ^a^	p.Gly44Ser	BC, 49 years	NR	NR
		c.657delC ^a^	p.Cys220AlafsTer15	BC, 34 years	0.00005270	1/2037
		c.2308C>T	p.Arg770Ter	BC, 44 years	0.00003567	2/2037
		c.2790C>T	p.(Lys931Serfs*14)	BC, 39 years	NR	NR
		c.2874C>G ^a^	p.Ser958Arg	BC, 48 years	0.0001085	2/2037
		c.2874C>G	p.Ser958Arg	BC, 78 years	0.0001085	2/2037

LoF: Loss of Function. ^a^ Previously found in Tavera-Tapia et al., 2019. ^b^ Age of diagnosis of breast cancer in the index cases sequenced. NR: not reported. BC = breast cancer.

### 2.6. Case-Control Association Study

The (likely) pathogenic/LoF variants found in 1993 BRCAX cases and ~50,000 gnomAD controls were compared. RStudio was used to obtain 95% Confidence Intervals and the Odds Ratio (OR), and an Exact Fisher Test was applied to obtain the significance of the association. A Bonferroni test was carried out as a multiple comparison test. For each gene, the analysis was carried out with the number of alleles carrying a likely pathogenic/LoF variant compared with the total number of wild-type alleles.

### 2.7. Combined Analysis

Regarding *RECQL5*, a combined analysis of our previous and current study was carried out, adding 700 samples from the first study to 1993 of the current and reaching a total of 2693 cases. All samples and selection of variants were performed by using identical criteria [31].

### 2.8. Splicing Studies

All missense and synonymous variants were analyzed by using the splicing module implemented in Alamut Visual 2.7.2. (SophiaGenetics, Lausanne, Switzerland) If an effect in splicing was predicted and RNA was available, functional assays were carried out to test the effect. RNA was isolated from heterozygote blood samples, and starting from 500 ng of RNA, we generated complementary DNA (cDNA) through RT-PCR. Primers were designed to test the predicted effect in splicing (available upon request), and PCR fragments were automatically sequenced in an ABI 3730xl instrument (Applied Biosystems, Waltham, MA, USA).

## 3. Results

### 3.1. RECQL1, BLM, WRN, and RECQL4 (Likely) Pathogenic Variants Are Not Associated with BC in Spanish BRCAX Cases

The (likely) pathogenic variants found in these RECQ helicase genes in the 1993 cases sequenced are shown in Table 1. All variants found were nonsense or frameshift, and all could be classified as pathogenic or likely pathogenic by following the ACMG guidelines [35]. We found two variants in *BLM*, one in *RECQL1*, seven in *RECQL4*, and five in *WRN*. Most of them were extremely rare and had not been reported in gnomAD or in the Spanish Variant Server database. We identified the variant c.1933C>T, p.Gln645Ter in *BLM*, which had previously appeared in the Australian and New Zealand population in association with BC [37]. Three of the seven variants found in the *RECQL4* gene, c.2161C>T and p.Arg721Ter, c.2269C>T and p.Gln757Ter, and c.2547_2548del and p.(Phe850Profs*33), had been previously described in the recessive syndromes associated to the gene [38,39].

Despite the rarity of the variants found, the case-control analysis comparing the (likely) pathogenic variants in these genes with pathogenic variants reported in gnomAD did not show any significant association. For the *RECQL4* and *WRN* genes, the number of variants was similar in cases and controls, while for *BLM* and *RECQL1*, the number of (likely) pathogenic variants was slightly lower among the cases. The OR and *p*-values for each association are shown in Table 3.

### 3.2. Analysis of RECQL5 (Likely) LoF Variants in 1993 BC-Only BRCAX Cases Shows a Tendency as a Moderate-Risk Gene Model

**Figure 1:**
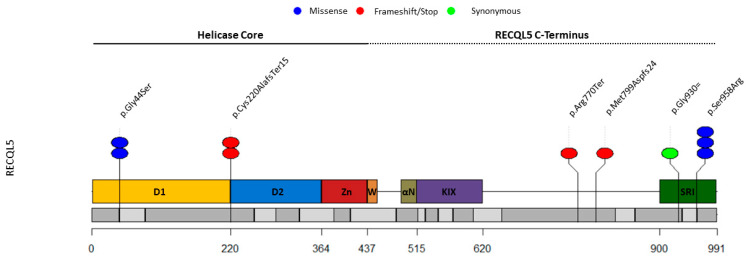
Map of the *RECQL5* protein domains, with circles indicating the location of the (likely) pathogenic germline variants identified in our current and previous studies (Table 2, Table 3 and Table 4). Colored boxes indicate the protein domains, and gray boxes the 19 coding exons of the *RECQL5* gene. The figure was created with RStudio (RStudio Team (2020). RStudio: Integrated Development for R. RStudio, PBC, Boston, MA, USA, URL http://www.rstudio.com/) (accessed on 25 February 2022). Figure adapted from Newman et al. [40].

After sequencing the 1993 BRCAX cases, we found five different (likely) LoF variants in *RECQL5* (Figure 1 and Table 2). Engagingly, three of the variants, c.130G>A and p.Gly44Ser, c.657delC and p.Cys220AlafsTer15, and c.2874C>G and p.Ser958Arg (detected twice in this study), had been found in our previous study and classified as LoF, LoF, and likely LoF, respectively [31]. We found another frameshift variant, c.2308C>T and p.Arg770Ter, and a synonymous variant, c.2790C>T, that was confirmed to generate an alternative splice donor-site at the end of exon 18, leading to the loss of the last 17 nucleotides of the exon and the generation of a premature stop codon in the penultimate coding exon of the gene (Supplementary Figure S1), and it was also classified as LoF.

In the case of *RECQL5*, the case-control study displayed an OR of 2.07 (*p* = 0.127; 95% CI, 0.74–4.74) (Exact Fisher Test), which, although not statistically significant, was in line with our previous results [31]. This led us to perform a combined analysis, adding the cases previously analyzed (Table 4) and achieving an OR of 2.56 (*p* = 0.009; 95% CI, 1.18–4.98) (Table 3), which is compatible with *RECQL5* as a moderate-risk susceptibility gene.

The phenotypes of the patients harboring LoF variants in *RECQL5* in the present and the previous study are shown in Table 2 and Table 4, respectively. All patients were diagnosed with BC; however, the mean age of diagnosis in the previous study was around ten years earlier (36.7 years of age) than that in the present study, i.e., 48.6 years of age. This is largely due to the older age of onset in one of the patients harboring the p.Ser958Arg variant who was diagnosed at 78 years of age.

Regarding the immunohistochemical profile of the tumors, in the previous study, considering all the LoF variants analyzed, we had found that half of the patients had developed a triple-negative tumor. In the present study, we had information available from two of the tumors and none of them was confirmed to be triple negative (Supplementary Table S1); however, for most of them, we did not have the information available; thus, we were not able to confirm or rule out our previous findings.

It is worth noting that, for the purpose of the combined analysis, we only considered those variants giving rise to a truncated protein. Although in Tavera-Tapia et al. [31], we had functionally characterized some of the missense variants found, we did not performed such a characterization in the present study, as our aim here was to increase the sample size, and we would not have been able to compare these types of variants with data from gnomAD.

### 3.3. Selection of Potentially Damaging Missense and Synonymous Variants in the RECQ Helicases

We made a selection of missense variants in the *RECQL1*, *BLM*, *WRN*, *RECQL4*, and *RECQL5* genes, which presented a likely pathogenic/LoF prediction (Supplementary Table S2) based on criteria previously described [31,36] that were not considered for the present analysis but could be selected for a further functional analysis.

The majority of the variants had not been previously reported, except for *RECQL1*, where half of the missense variants selected (4/8) had been reported in Dorling et al., 2021 [3]. It is worth noting that, in that study, it is suggested that missense variants in *RECQL1* might show a marginal association with BC risk (OR, 1.12; *p* = 0.047; 95% CI, 1.00–1.26); however, given that that study, like ours, lacks a functional characterization of the missense variants, we do not believe that these results are valuable.

## 4. Discussion

In this study, we sought to shed light on the uncertainty about whether any of the members of the RECQ helicase family (*RECQL1*, *BLM*, *WRN*, *RECQL4*, and *RECQL5*) could have a role in BC susceptibility. Numerous studies have proven the relation of these helicases with key cellular pathways, such as DNA repair, recombination, replication, transcription, telomere maintenance, and mitochondrial function [10]. Biallelic mutations in all of them, except for *RECQL5*, are related to chromosome instability and cancer-predisposition syndromes, including the recently described RECON syndrome [16,24,25,28]. Several studies have pointed out these helicases, especially *RECQL1* and *RECQL5*, as new BC susceptibility genes; however, whether any of them have a role is still under debate [3,15,22,31,41,42]. In an attempt to clear the doubts about the involvement of the RECQ helicases in this disease, we sequenced the whole coding sequence of the five genes in 1993 BRCAX Spanish patients and compared the results with approximately 50,000 control individuals from gnomAD.

Regarding *RECQL1*, *BLM*, *WRN*, and *RECQL4*, we did not obtain a significant association or a trend of association, except for *RECQL1*, for which we obtained an OR of 0.20 with a marginal *p*-value (*p* = 0.097; 95% CI, 0.005–1.144), which could make us think about a protective effect. These results are in line with those found in the largest case-control study so far [3], where the number of Protein-Truncating variants found in the cases was slightly lower than in the controls; however, the association was not statistically significant (OR, 0.84; *p* = 0.21; 95% CI, 0.64–1.10). Given the much larger sample size analyzed in Dorling et al. (2021), we do not think that the trend that we observed really reflects a putative protective effect; however, it at least supports a lack of association of *RECQL1* with an increased BC risk.

In the case of *BLM*, we did not find a significant association, and this is in line with a large study performed in a series of almost 20,000 Polish BC patients and controls that was recently published [23]. In the case of *WRN* and *RECQL4*, previous studies had suggested the possibility of these as susceptibility genes, but the cohorts were small [26,27,29,30]; to our knowledge, our cohort is the largest to date. Scrutinizing our findings, we conclude that none of these four genes has a major role in BC susceptibility, at least in the Spanish population, but further studies are needed in order to rule them out definitely.

However, in the case of *RECQL5*, the outcome was different and encouraging, given that, within our 1993 samples, we found six LoF or likely LoF variants (Table 2) obtaining an OR of 2.07 (*p* = 0.127; 95% CI, 0.76–4.89), thus confirming its tendency as BC-susceptibility gene (Tavera-Tapia et al., 2019). Moreover, from the five different (likely) LoF variants identified in our study, three of them, namely c.130G>A and p.Gly44Ser, c.657delC and p.Cys220AlafsTer15, and c.2874C>G and p.Ser958Arg, were detected twice in this study (Table 4) [31]. Interestingly, one of the LoF variants characterized in this study is a synonymous change, c.2790C>T, that turned out to alter splicing and generate a PTC (Supplementary Figure S1). This highlights the importance of making a careful revision of the silent changes that, in some studies, are systematically classified as (likely) benign without further analysis. In this case, the c.2790C>T variant was predicted to generate a cryptic splice-donor site by the splicing module integrated in the software Alamut Visual Plus version v1.4 that, together with its absence in gnomAD, led us to perform a functional characterization that confirmed its effect in splicing.

In our previous study, we had found seven (likely) LoF variants in 700 samples and only one in 665 controls sequenced, which brought us an OR of 6.7 (*p* = 0.07; 95% CI, 0.95–302.75). On the other hand, considering only Protein-Truncating variants, and comparing with gnomAD non-cancer European non-Finnish individuals, we found almost four times more truncated variants in our cases, obtaining an OR of 3.99 (*p* = 0.02; 95% CI, 1.05–10.70). Given that the present study showed the same tendency as our previous one, we decided to combine both studies, and this allowed us to achieve 2693 BC cases, harboring 10 (likely) LoF (Table 2 and Table 4) and reaching an OR of 2.56 (*p* = 0.007; 95% CI, 1.21 to 5.15), which, in clear contrast to the rest of helicases, reinforces the position of *RECQL5* as a candidate moderate-susceptibility gene in BC.

In is worth noting that, in the combined study, the c.657delC and p.Cys220AlafsTer15 and the c.130G>A and p.Gly44Ser variants appeared twice each, while c.2874C>G and p.Ser958Arg appeared three times. Furthermore, the two first variants did not appear in gnomAD or the Spanish Variant Server. This may lead us to think about a population-based effect, as seen with variants in other BC-susceptibility genes, such as *BRCA1*, *BRCA2*, or *BARD1* [43,44]; however, further clarification is needed.

Regarding the phenotype of the patients, the mean age of the first BC diagnostic was 48.6 years old in this study and 42.7 years old in the combined study, similar to *BRCA1* and *BRCA2* in the Spanish population, with an average of 43.6 and 42.8 years old, respectively [45]. In our previous study, we had found that half of the patients (3/6) harboring LoF variants in *RECQL5* had developed a triple-negative BC, and we speculated that they could be associated with a more severe phenotype [31]. However, the lack of information of the immunohistochemistry information in the present study (only available for two of the six patients) did not allow us to confirm these results (Supplementary Table S1).

Finally, we also found a series of missense variants that were classified as candidates for further analysis, based on the LoF prediction obtained with a combination of predictors, as previously described [31,36]. Although in this study we did not perform a functional characterization of these variants, we and others have found that some missense variants in the gene can affect the helicase activity and other functions of the protein [31,40] and turn out to be LoF. The lack of functional studies may lead to the underestimation of our current outcome.

## 5. Conclusions

In summary, the present results, together with our previous study, place *RECQL5* as the only RECQ helicase showing a significant association with BC susceptibility. Although larger studies are needed before translating these to the clinics, we believe that our findings are encouraging enough to boost further analysis of *RECQL5* as a new BC candidate susceptibility gene, including a functional analysis to help unravel the significance of missense variants in the gene.

## Figures and Tables

**Table 3:** Cases-control analysis of the variants found in the five helicase genes analyzed.

Gene	Cases-Heterozygotes/Non-Heterozygotes	Controls-Heterozygotes/Non-Heterozygotes ^a^	Odds Ratio	*p*-Value	Confidence Interval
***RECQL1***	1/1992	132/51,061 ^b^	0.20	0.097	0.01–1.14
***BLM***	2/1991	125/51,199	0.42	0.338	0.05–1.55
***WRN***	5/1988	113/51,180	1.15	0.628	0.37–2.77
***RECQL4***	9/1984	209/50,447	1.10	0.721	0.50–2.13
***RECQL5***	6/1987	74/50,883	2.07	0.127	0.74–4.74
***RECQL5* ^c^**	10/2683	74/50,883	2.56	0.009	1.18–4.98

Heterozygotes = number of individuals carrying (likely) LoF variants. ^a^ In *RECQL5* controls, variant c.2874C>G and p.Ser958Arg was considered, although it was not flagged as LoF in gnomAD, as it was considered as a likely LoF in the cases. ^b^ The total number of controls came from the median of the total number of alleles obtained for each gene in gnomAD. ^c^ Combined analysis of *RECQL5* adding data from Tavera-Tapia et al., 2019 [31].

**Table 4:** *RECQL5* LoF protein-truncating variants found in 700 samples in Tavera-Tapia and added to the current study.

Gene	Reference	Nucleotide Change	Protein Change	Phenotype	gnomAD	CSVS
***RECQL5***	NM_004259.6	c.130G>A	p.Gly44Ser	BC, 50 years	NR	NR
		c.657delC	p.Cys220AlafsTer15	BiBC, 34 years, 46 years	0.00005270	1/2037
		c.2393dupC	p.Met799Aspfs*24	BiBC, 37 years, 39 years	NR	NR
		c.2874C>G	p.Ser958Arg	BC, 26 years	0.0001085	2/2037

BC, breast cancer; BiBC, bilateral BC.

## Data Availability

Variants reported in this study were submitted to LOVD v.3.0—Leiden Open Variation Database (https://www.lovd.nl/) with the following variant accession numbers: (*RECQL5*) 0000873660, 0000873661, 0000873663, 0000873666, and 0000873668; (*RECQL1*) 0000873675; (*BLM*) 0000873682 and 0000873684; (*WRN*) 0000873694, 0000873695, 0000873696, 0000873697, and 0000873698; (*RECQL4*) 0000873699, 0000873700, 0000873701, 0000873702, 0000873703, 0000873712, and 0000873713. All primer sequences used for Sanger validation of the variants are available upon request.

## Supplementary Materials

The following supporting information can be downloaded at: https://www.mdpi.com/article/10.3390/cancers14194738/s1. Figure S1: Sanger sequencing of the patient harboring the c.2790C>T; Gly930 = synonymous variant in *RECQL5* at cDNA level. Table S1: Breast tumor immunohistochemical features of the *RECQL5* LoF variant heterozygotes. Table S2: Missense and synonymous variants with likely LoF prediction gathered in the 1993 samples.
