# Supplementary material for: A Large Case-Control Study Performed in Spanish Population Suggests That RECQL5 Is the Only RECQ Helicase Involved in Breast Cancer Susceptibility
Source: Cancers (Basel). 2022 Sep 28;14(19):4738. doi: 10.3390/cancers14194738 (PMC9563930; doi:10.3390/cancers14194738)
Supplement: Supplementary file 1 [file cancers-14-04738-s001.zip › Figure S1 Cancers.pdf]

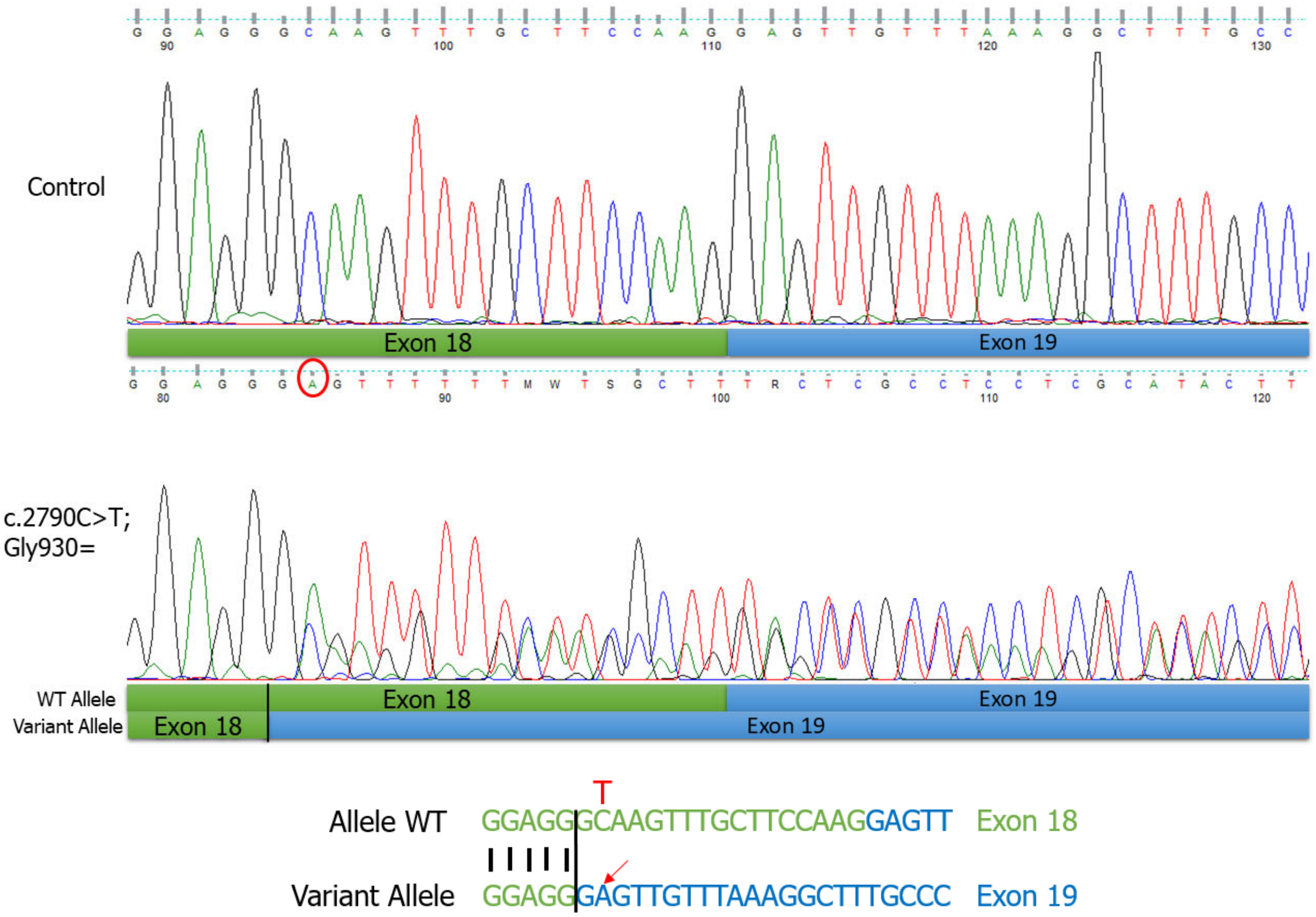

Figure S1. Sanger sequencing of the patient harboring the c.2790C>T; Gly930= synonymous variant in RECQL5 at cDNA level (lower figure) and a control sample (upper figure). The circle and arrow in red indicate the point where the change is located. The C>T change generates a GT cryptic splicing donor site at the end of exon 18, leading to the cut before these two nucleotide and generating a loss of the last 17 nucleotides of the exon and the generation of a premature stop codon in the penultimate coding exon of the gene (p.(Lys931Serfs*14).
