# Supplementary material for: A Large Case-Control Study Performed in Spanish Population Suggests That RECQL5 Is the Only RECQ Helicase Involved in Breast Cancer Susceptibility
Source: Cancers (Basel). 2022 Sep 28;14(19):4738. doi: 10.3390/cancers14194738 (PMC9563930; doi:10.3390/cancers14194738)
Supplement: Supplementary file 1 [file cancers-14-04738-s001.zip › Table S1 Cancers.pdf]

Table S1. Breast tumor immunohistochemical features of the *RECQL5* LoF variant heterozygotes.

Mutation	Type of tumor	Age of onset	ER status	PR status	Her2 status
p.Gly44Ser	IDC	49	NA	NA	NA
p.Gly44Ser[a]	NA	50	NA	NA	NA
p.Ser958Arg[a]	NA	26	Positive	Positive	NA
p.Ser958Arg	IDC	48	Positive	Positive	Negative
p.Ser958Arg	NA	78	NA	NA	NA
c.2790C>T; (p.=)	DCIS	39	Negative	Negative	NA
p.Arg770Ter	NA	44	NA	NA	NA
p.Cys220AlafsTer15	ILC+IDC	34	NA	NA	NA
p.Cys220AlafsTer15[a]	NA	34	Negative	Negative	Negative
p.Met799Aspfs*24[a]	NA	37	Negative	Negative	Negative

[a]Variants from Tavera-Tapia 2019. NA, Not Available. ER=estrogen receptor. PR=Progesterone receptor. IDC=Invasive Ductal Carcinoma. DCSI=Ductal Carcinoma *in situ*. ILC= Invasive Lobulillar Carcinoma.
