# Supplementary material for: A Large Case-Control Study Performed in Spanish Population Suggests That RECQL5 Is the Only RECQ Helicase Involved in Breast Cancer Susceptibility
Source: Cancers (Basel). 2022 Sep 28;14(19):4738. doi: 10.3390/cancers14194738 (PMC9563930; doi:10.3390/cancers14194738)
Supplement: Supplementary file 1 [file cancers-14-04738-s001.zip › Table S2 Cancers.pdf]

Table S2. Missense and synonymous variants with likely LoF prediction gathered in the 1993 samples.

Gene	Nucleotide Change	Protein Change	P	M	SG	MA	F	C	MP	S	PPS	Prediction	Splicing	Varsome	Franklin	Previously Found
RECQL	c.385T>G	p.Cys129Gly	D	D	D	T	T	D	D	D	T	YES	NO	VUS (PM2, PP3)	VUS (PM2)	[3]
	c.386G>A	p.Cys129Tyr	D	D	D	T	T	D	D	D	T	YES	NO	VUS (PM2, PP3)	VUS (PM2)	[3]
	c.401C>T	p.Thr134Ile	D	D	T	D	T	D	D	D	T	YES	NO	VUS (PM2, PP3)	PB (BS1, BP6)	NR
	c.1643A>C	p.His548Pro	D	D	D	D	T	D	D	D	D	YES	NO	VUS (PM2)	VUS (PM2)	NR
	c.1184T>C	p.Met395Thr	D	D	D	T	T	D	T	D	T	YES	NO	VUS (PM2, PP3)	VUS (PM2)	[3]
	c.49G>A	p.Glu17Lys	D	T	T	D	T	D	D	D	T	YES	YES	VUS (PM2, PP3)	VUS (PM2)	[3]
	c.863A>T	p.Tyr288Phe	D	D	T	T	T	D	D	D	T	YES	NO	VUS (PM2, PP3)	VUS (PM2, PP3)	NR
BLM	c.1211G>A	p.Arg404Gln	D	T	D	D	T	D	D	D	T	YES	NO	VUS (PM2)	VUS (PM2)	NR
	c.2498C>T	p.Ala833Val	D	D	D	D	T	D	D	D	D	YES	NO	VUS (PM2, PP3)	VUS (PM2)	NR
	c.47A>G	p.His16Arg	D	T	D	D	T	D	T	D	D	YES	NO	VUS (PM2, PP3)	VUS (PM2)	NR
	c.2909T>C	p.Val970Ala	D	D	D	T	T	D	T	D	T	YES	NO	VUS (PM2)	VUS (PM2)	NR
	c.2474C>T	p.Pro825Leu	D	D	D	D	T	T	D	D	D	YES	NO	VUS (PM2, PP3)	VUS (PM2)	NR
	c.4240T>C	p.Tyr1414His	D	T	T	D	T	D	T	D	D	YES	NO	VUS (PM2)	VUS (PM2)	NR
	c.2141C>G	p.Ser714Cys	D	D	D	D	T	D	D	D	D	YES	NO	VUS (PM2, PP3)	VUS (PM2, PP3)	NR
	c.475G>A	p.Asp159Asn	D	T	D	D	T	D	D	D	T	YES	NO	VUS (PM2, PP3)	VUS (PM2)	NR
WRN	c.2923G>A	p.Glu975Lys	D	T	D	D	T	D	T	T	D	YES	NO	VUS (PM2, BP4)	VUS (PM2, BP4)	NR
	c.2822C>A	p.Ser941Tyr	D	T	T	D	T	T	T	T	T	NO	YES	VUS (PM2, BP4)	VUS (PM2, BP4)	NR
	c.2029G>A	p.Gly677Arg	D	T	D	D	T	D	D	D	D	YES	NO	VUS (PM2, PP3)	VUS (PM2, BS2)	NR
	c.2034T>A	p.His678Gln	D	D	D	D	T	D	D	D	T	YES	NO	VUS (PM2, PP3)	VUS (PM2)	NR
	c.3003C>G	p.His1001Gln	D	T	T	D	T	T	D	D	D	YES	NO	VUS (PM2, BP4)	VUS (PM2)	NR
	c.2132G>A	p.Arg711Gln	D	T	D	T	T	T	T	D	D	NO	YES	VUS (PM2, BP4)	VUS (PM2, BP4)	NR
	c.1455C>T	p.=	-	-	-	-	-	-	-	-	-	NO	YES	PB (BP4, BP7)	VUS (PM2, BP7)	NR
RECQL4	c.2351G>A	Arg784Gln	D	D	T	D	T	T	D	D	D	YES	NO	VUS (PM2)	VUS (PM2)	NR
	c.2425G>A	p.Gly809Arg	D	T	D	D	T	T	D	D	D	YES	NO	VUS (PM2)	VUS (PM2)	NR
	c.2362C>T	p.His788Tyr	D	D	D	D	T	T	D	D	D	YES	NO	VUS (PM2, PP3)	VUS (PM2)	NR

	c.1853G>A	p.Arg618Gln	D	D	D	T	T	T	T	D	D	YES	NO	VUS (PM2)	VUS (PM2)	NR
	c.2765C>T	Thr922Ile	D	D	D	D	T	T	D	D	D	YES	NO	VUS (PM2)	VUS (PM2)	NR
RECQL5	c.1712C>G	p.Ala571Gly	T	D	T	T	T	T	T	T	T	NO	YES	VUS (PM2, BP4)	VUS (PM2, BP4)	NR
	c.130G>A	p.Gly44Ser	T	T	D	D	T	D	T	T	T	NO	YES	PP (PM2, PP3)	VUS (PM2, PP3)	NR
	c.1534A>G	p.Met512Val	D	T	T	D	T	T	T	T	T	NO	YES	VUS-PP (PM2, PP3)	VUS (PM2)	NR
	c.569C>G	p.Ala190Gly	D	D	T	D	T	T	D	D	D	YES	NO	VUS-PP (PM2)	VUS (PM2)	NR
	c.1601T>C	p.Leu534Pro	D	D	D	D	T	D	D	D	D	YES	NO	VUS-PP (PM2, PP3)	VUS (PM2)	NR
	c.2926C>T	p.Arg976Trp	D	T	D	D	T	T	T	D	D	YES	NO	VUS (PM2)	VUS (PM2)	NR
	c.1648C>T	p.Arg550Trp	D	T	D	T	T	D	D	D	D	YES	NO	VUS (PM2, PP3)	VUS (PM2)	NR
	c.305C>T	p.Ser102Leu	D	T	T	T	T	D	D	D	D	YES	NO	VUS-PP (PM2, PP3)	VUS (PM2)	NR
	c.1564A>G	p.Ile522Val	D	T	T	D	T	D	T	D	T	NO	YES	VUS (PM2, BP4)	VUS (PM2, BP4)	NR

P: polyphen-2; M: MUTTASTER; SG: SNPs&Go; MA: MutationAssesor; F: FATHMM; C: Condel; MP: MutPred; S: SIFT; D: Deleterious; T: Tolerated; PPS Score was calculated with SNAP2 algorithm (https://www.predictprotein.org/). Scores >50 were considered likely LoF. Prediction: YES: Missense variant considered likely LoF by at least 5/8 predictors. Splicing: YES: based on the splicing module integrated in Alamut Visual 2.7.2. Varsome and Franklin prediction of pathogenicity based on ACMG guidelines. VUS: Variant of Unknown Significance; PP: Probably Pathogenic; PB: Probably Benign. PM2: absent variant in controls or a steeply low frequency in Exome Sequencing Project, 1000 Genomes or ExAC. PP3: pathogenic evidence demonstrated from models and computational predictions. BP4: benign effect proven through models and computational predictions. BP6: reported variants as benign, but with not enough evidence to evaluate them independently. BP7: synonymous variant with negative splicing alteration prediction from each platform. Strong benign evidence: BS1: allele frequency higher than expected in variants involved in diseases. BS2: variant observed in a healthy adult and in a recessive illness with complete penetrance.
